# Enhancing breast cancer outcomes with machine learning-driven glutamine metabolic reprogramming signature

**DOI:** 10.3389/fimmu.2024.1369289

**Published:** 2024-05-01

**Authors:** Xukui Li, Xue Li, Bin Yang, Songyang Sun, Shu Wang, Fuxun Yu, Tao Wang

**Affiliations:** ^1^ Research Laboratory Center, Guizhou Provincial People’s Hospital, Guiyang, Guizhou, China; ^2^ NHC Key Laboratory of Pulmonary Immune-related Diseases, Guizhou Provincial People’s Hospital, Guizhou University, Guiyang, Guizhou, China; ^3^ Department of Breast Surgery, Guizhou Provincial People’s Hospital, Guiyang, Guizhou, China

**Keywords:** breast cancer, glutamine metabolism programming, prognosis, immunotherapy, BI-2536

## Abstract

**Background:**

This study aims to identify precise biomarkers for breast cancer to improve patient outcomes, addressing the limitations of traditional staging in predicting treatment responses.

**Methods:**

Our analysis encompassed data from over 7,000 breast cancer patients across 14 datasets, which included in-house clinical data and single-cell data from 8 patients (totaling 43,766 cells). We utilized an integrative approach, applying 10 machine learning algorithms in 54 unique combinations to analyze 100 existing breast cancer signatures. Immunohistochemistry assays were performed for empirical validation. The study also investigated potential immunotherapies and chemotherapies.

**Results:**

Our research identified five consistent glutamine metabolic reprogramming (GMR)-related genes from multi-center cohorts, forming the foundation of a novel GMR-model. This model demonstrated superior accuracy in predicting recurrence and mortality risks compared to existing clinical and molecular features. Patients classified as high-risk by the model exhibited poorer outcomes. IHC validation in 30 patients reinforced these findings, suggesting the model’s broad applicability. Intriguingly, the model indicates a differential therapeutic response: low-risk patients may benefit more from immunotherapy, whereas high-risk patients showed sensitivity to specific chemotherapies like BI-2536 and ispinesib.

**Conclusions:**

The GMR-model marks a significant leap forward in breast cancer prognosis and the personalization of treatment strategies, offering vital insights for the effective management of diverse breast cancer patient populations.

## Introduction

Breast cancer (BC) remains the most prevalent malignant tumor among women worldwide, leading to the highest number of cancer-related deaths in this population (1). In 2022, the estimated incidence of BC reached approximately 2.26 million cases globally, underscoring its significant impact on women’s health (2). Given its rising prevalence, particularly in developing countries like China, there is a pressing need for improved diagnostic, treatment, and prognostic strategies for BC. BC is categorized into non-invasive and invasive types, with the latter’s ability to metastasize to distant organs such as the bones, liver, lungs, and brain, often rendering it incurable (3). Despite advancements in treatment technologies, the prognosis for late-stage BC patients remains dismal (4). Thus, developing an effective predictive model is crucial for enhancing treatment outcomes for BC patients.

Metabolic reprogramming is pivotal in tumor development, exemplified by the Warburg effect, which highlights the crucial role of cancer cell metabolism in supporting cancer survival and proliferation (5). This metabolic reprogramming is characterized by the preferential conversion of glucose to lactate under aerobic conditions (6). Beyond glucose, glutamine is also a significant contributor to redox balance, essential for the metabolic reconfiguration of tumor cells (7). As a versatile amino acid abundant in the human bloodstream, glutamine supports various metabolic processes. It contributes to the synthesis of purines and pyrimidines, participates in the tricarboxylic acid cycle, and supports the biosynthesis of hexosamines, nucleotides, and asparagine, besides being a critical respiratory fuel for tumor cells (8). The aberrant metabolism of glutamine is increasingly recognized as a vital component of BC cell survival and growth (9). Research suggests that glutamine metabolism reprogramming (GMR) within the tumor microenvironment (TME) may significantly affect the anti-tumor immune response (7). Given glutamine’s essential role in supporting oxidative metabolism in certain cancer cell lines, investigating glutamine metabolism abnormalities in BC is of paramount importance for improving treatment and prognosis (10). This study aims to develop a novel prognostic model centered on GMR to enhance BC patients outcomes.

## Methods and materials

### Data acquisition

The dataset for training was meticulously assembled from the TCGA database, encompassing gene profiles, mutational data, and clinical information pertinent to breast cancer cases. We ensured the inclusion of samples with available survival data, guaranteeing dataset completeness and accuracy.

To strengthen and validate our findings, we sourced additional datasets from the GEO database, specifically from studies GSE93601, GSE76250, GSE70947, GSE202203, GSE96058, GSE58812, GSE21653, GSE86166, GSE20685, GSE20711, GSE88770, GSE6532. This approach allowed for cross-validation of our results across diverse datasets, enhancing the reliability of our findings.

### Single-cell sequencing technique

Our study utilized single-cell data from the GEO database (GSE161529) (11). The initial step involved discarding genes with zero expression levels, retaining those with non-zero expression. We normalized the expression matrix using the “SC Transform” function from the Seurat R package. Dimensionality reduction was achieved through PCA and UMAP methods, followed by cell cluster identification using the “FindNeighbors” and “Find Clusters” functions. The DoubletFinder R package helped in eliminating doublets, ensuring data accuracy (12). Cells with either 15% mitochondrial gene content or fewer than 500 genes were excluded, refining the dataset further.

Post-quality control, approximately 43,766 cells were preserved for in-depth analyses. Celltypist facilitated the categorization of cell types, laying a robust foundation for our research (13). Tumor cells were identified using the copyKAT algorithm (14).

### Cell-cell communication analysis

We generated CellChat objects for each group using the “CellChat” R package, with “CellChatDB.human” as our reference database (15). All analyses utilized default parameters. To compare interaction counts and intensities across groups, we amalgamated CellChat objects via the “mergeCellChat” function. Differences in cell interactions and signaling pathways were visualized and analyzed using specific functions.

### Functional analysis

We utilized the GO and KEGG databases for a comprehensive evaluation of differential GMR-related gene expression between tumor and normal tissues (16, 17). The Enrichplot package and clusterProfiler algorithm facilitated this analysis, with a focus on Gene Set Enrichment Analysis between distinct risk subgroups (18). A False Discovery Rate below 0.05 was considered significant.

### Calculating the GMR-score

We utilized the TCGA-BRCA dataset to conduct a differential gene expression analysis between tumor and normal breast tissues. This analysis allowed us to identify a set of 67 genes with differential expression associated with glutamine metabolism. Our development of the GMR-score integrated these gene expressions, drawing from a selection of genes determined through the GeneCards database with a relevance score exceeding the threshold of 8. The heatmap and network generated as a result visualized not only the expression but also the interconnections among these GMR genes. We applied the ssGSEA and Ucell algorithms to bulk and single-cell data to compute the GMR-score, which provided an indirect estimation of glutamine metabolism-related gene activity within the breast cancer tissues (19, 20). It is crucial to emphasize that the GMR-score, while designed to estimate glutamine metabolic activity and provide insights into the metabolic adaptations within the tumor microenvironment, does not directly measure metabolic flux. Instead, it functions as an estimator, based on gene expression data indicative of glutamine metabolic pathways, enabling us to distinguish between the gene expression profiles of tumor versus normal tissue, and offering a potential link to the metabolic state of the tumor. Spearman analysis elucidated the association between GMR-score and immune cell infiltrations, providing a comprehensive evaluation of the GMR-score in breast cancer.

### Construction of the GMR model and nomogram

To establish a GMR-based prognostic model specifically tailored for BC patients, we employed a comprehensive workflow initially proposed by Liu et al. (21). This approach integrated a diverse set of ten computational algorithms, including Random Forest (RSF), LASSO (Least Absolute Shrinkage and Selection Operator), Gradient Boosting Machine (GBM), Survival-SVM (Support Vector Machine), SuperPC (Supervised Principal Components), Ridge Regression, plsRcox (Partial Least Squares Regression for Cox’s model), CoxBoost, Stepwise Cox, and Elastic Net (Enet). Notably, RSF, LASSO, CoxBoost, and Stepwise Cox play critical roles in reducing dimensionality and selecting relevant variables.

Utilizing the TCGA-BRCA dataset as our training cohort, we applied these algorithms to generate a prognostic signature. Subsequently, we evaluated the performance of the model across all available cohorts, which included TCGA and five external datasets, by calculating the average concordance index (C-index). This metric served as a measure of the model’s discriminative ability, and through this process, we were able to identify the most consistent and reliable prognostic model for BC.

The formulation of the GMR-model stands as a pivotal outcome of this research, offering a valuable tool for assessing BC outcomes. The model’s robustness and accuracy were thoroughly validated through various methods, including calibration curves, decision curve analysis (DCA), and multivariate Cox regression analyses. These validation steps were instrumental in confirming the relevance and association of the identified prognostic GMR genes with BC. The risk scores for individual patients were calculated using the following formula:


$$riskscore\;=\;\sum_{i=1}^{n}\left(\beta_{i}\times Exp_{i}\right)$$

In this equation, ‘n’ represents the total number of GMR genes included in the model, ‘Exp’ denotes the expression levels of the GMR genes, and ‘β’ signifies the coefficients derived from the multivariate Cox regression model. Based on the calculated risk scores, patients were stratified into high-risk and low-risk groups.

To validate the generalizability and reliability of our GMR-model, we utilized several external datasets, ensuring that our findings were consistent beyond the initial TCGA training set. The survival disparities between the high-risk and low-risk groups were assessed using Kaplan-Meier (KM) survival analysis, with a p-value less than 0.05 considered statistically significant. This step was crucial to confirm the prognostic value of the GMR-model in different patient cohorts and settings.

### Genomic alteration analysis

To delineate genetic disparities between high- and low-risk BC subgroups, we conducted a comprehensive analysis of genetic mutation frequencies and copy number alterations (CNA) utilizing data from the TCGA-BRCA database.

We calculated the tumor mutation burden (TMB) for each subgroup, extracting the relevant data from raw mutation files. Utilizing maftools, we subsequently mapped the mutation landscape, focusing on the 28 genes (mutation rate > 5%) that exhibited the highest mutation frequencies. Following procedures outlined in a previously published study (22), we utilized the deconstructSigs package to identify patient-specific mutational signatures. This analysis brought to light four prominent mutational signatures (SBS1, SBS3, SB11, SBS12) within the BC dataset, all of which demonstrated elevated mutation frequencies.

In addition to these mutational signatures, our investigation extended to chromosomal aberrations. We pinpointed the five regions most frequently subjected to amplification and deletion events, giving special attention to four predominant genes located in chromosomal regions 8q24.21 and 9p23. This meticulous analysis aims to uncover the genetic underpinnings that may contribute to the variance in risk and prognosis between the two BC patient subgroups.

### Identifying TME disparities

To assess immune cell infiltration levels precisely and exhaustively, we analyzed the prevalence of adversely infiltrated immune cells in patients categorized by the GMR-model. IOBR package employes a comprehensive suite of algorithms, including MCPcounter, EPIC, xCell, CIBERSORT, quanTIseq, and TIMER, ensuring a robust and multifaceted examination (23–29).

In addition, we evaluated the ESTIMATE and TIDE indices to glean insights into the immune microenvironment’s state and structure within the TME (30, 31). This evaluation is crucial, as it provides valuable information for immunotherapy strategies and enhances our understanding of the potential outcomes for BC patients.

To augment our analysis, we also quantified immune checkpoints, thereby offering an additional layer of insight into the immune state. This quantification serves as a preliminary tool for predicting patient responsiveness to immune checkpoint inhibitors (ICIs) therapy, a vital component in personalized cancer treatment strategies.

### Determining therapeutic targets and drugs

After removing duplicates, we gathered a comprehensive collection of 6,125 compounds from the Drug Repurposing Hub (https://clue.io/repurposing). Our objective was to predict chemotherapeutic responses and identify potential therapeutic targets. The selection of these targets, associated with BC outcomes, was based on Spearman correlation analysis. Specifically, we looked at the relationship between risk scores and gene expression levels, focusing on cases where the correlation coefficient exceeded 0.15 and the P-value was less than 0.05. To pinpoint genes associated with adverse prognosis (correlation coefficient < -0.15, P < 0.05), we examined the relationship between CERES scores and risk scores for brain cells using data from the Cancer Cell Line Encyclopedia (CCLE) (32).

To refine our drug response predictions, we employed the CTRP and PRISM datasets, both of which provide extensive drug screening and molecular information across various cancer cell lines. Our analysis included differential expression assessments between bulk samples and cell lines. Utilizing the pRRophetic package, we applied a ridge regression model for predicting drug responses. This model, trained using expression data and drug response information from solid cancer cell lines, exhibited robust performance, as validated through standard 10-fold cross-validation (33).

Furthermore, we conducted a Connectivity Map (CMap) analysis to identify the most promising therapeutic drugs for BC. This involved comparing gene expression profiles between different risk subgroups and submitting the top 300 genes (comprising 150 up-regulated and 150 down-regulated genes) to the CMap platform (https://clue.io/query). Interestingly, the CMap scores showed a negative correlation with the potential therapeutic efficacy in BC, providing valuable insights for drug selection.

### Immunohistochemistry experiment

In our study, we collected tissue samples from 30 BC patients undergoing surgery at the Guizhou Provincial People’s Hospital. These samples were then processed through Hematoxylin and Eosin (HE) staining, adhering to standard protocols. The diagnostic evaluations of these stained specimens were carried out by two independent pathologists, ensuring an unbiased and thorough analysis. Detailed demographic and clinical information of the patient cohort is provided in **Supplementary Table S1**.

Furthermore, we conducted immunohistochemistry (IHC) on the paraffin-embedded tissue specimens, employing methodologies that have been outlined in previous publications (34, 35). The antibodies utilized in this process are comprehensively listed in **Supplementary Table S2**. The evaluation of the protein expression levels was conducted following well-established protocols and scoring criteria. Consistent with the practices established in our prior research (35), the assessment was independently carried out by two pathologists, ensuring a reliable and accurate interpretation of the protein expression levels.

## Results

### Deciphering the impact of GMR-related genes in BC

**Figure 1:**
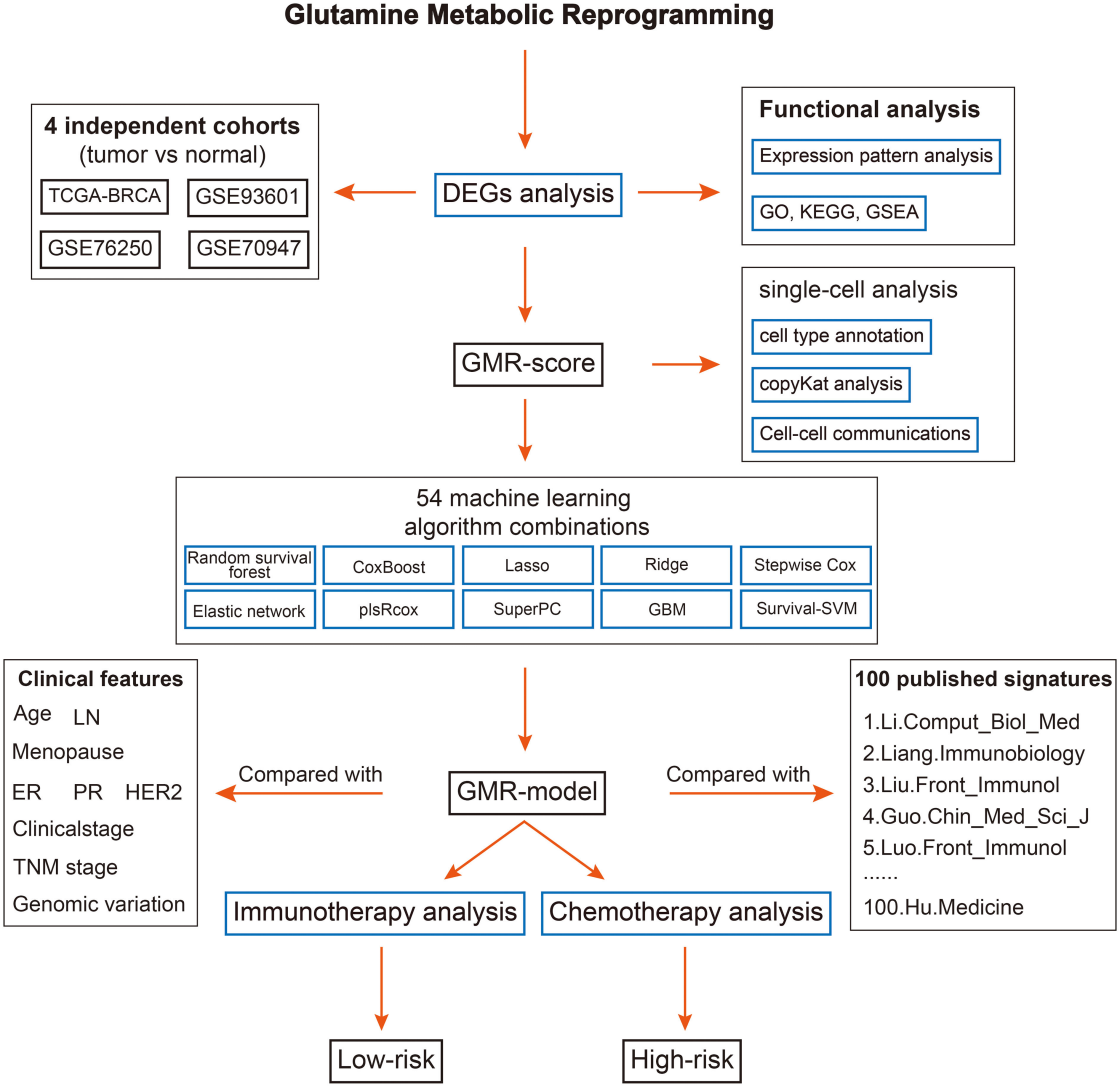
The overall flow of this study.

**Figure 2:**
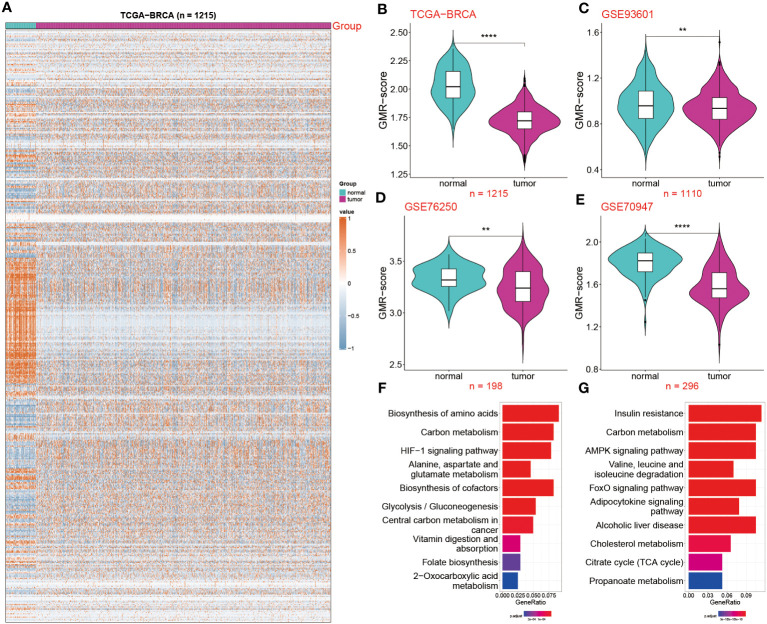
Deciphering the impact of GMR-related genes in BC. **(A)** Expression profile of GMR related regulators in breast cancer and normal tissues. **(B–E)** The GMR-score was compared among two groups in the four datasets: TCGA-BRCA, GSE93601 GSE76250 and GSE70947. **(F, G)** KEGG enrichment analysis of the up and down-regulated GMR genes. **P < 0.01, ****P < 0.0001.

The overall design of this study is displayed in **Figure 1**. In this study, we used TCGA-BRCA database to screen differentially expressed genes (DEGs) of GMR between tumor and normal samples. Significant DEGs were shown in the heatmap (**Figure 2A**). To systematically clarify the relationship between prognostic DEGs, we categorized them into three groups and constructed a regulatory network illustrating their interactions, with most showing significant associations (**Supplementary Figure S1A**). Further exploring the link between GMR genes and BC, we developed a GMR-score, based on the prognostic DEGs associated with glutamine metabolism. The GMR score was computed using the ssGSEA and Ucell algorithms for bulk and single-cell data, respectively. These algorithms allowed us to quantitatively estimate the activity level of glutamine metabolism-related genes in individual samples, providing an indirect measure of glutamine metabolism activity within the tumor microenvironment. This analysis revealed that the GMR-scores were markedly lower compared to control cohorts (**Figure 2B**), a finding corroborated by three additional datasets (GSE93601, GSE76250, and GSE70947) (**Figures 2C–E**). These results underscore the critical role of GMR-related gene expression differences in BC development. Subsequently, we delved deeper into the functions and pathways of these DEGs. The KEGG results showed that pathways related to biosynthesis of amino acids, carbon metabolism, and HIF-1 signaling pathway show a higher gene ratio, suggesting these pathways are upregulated in the tumor environment and may contribute to tumorigenesis (**Figure 2F**). However, pathways such as AMPK signaling pathway, and Valine, leucine and isoleucine degradation were most inhibited. The downregulation of these pathways could reflect a suppression of normal metabolic processes in tumor tissues (**Figure 2G**).

Extensive research by scholars on tumors consistently suggests that the TME directly influences tumor onset and progression. Building on this, we delved into the correlation patterns between the GMR-score and 25 types of infiltrating immune cells (**Supplementary Figure S1B**). Notably, multiple cells, including Th1 cells, resting dendritic cells, resting CD4 memory T cells, and Th17 cells, exhibit a positive correlation with the GMR-score, while T cells follicular helper, memory B cells, plasma cells are negatively correlated. In addition, we also demonstrated the strongest positive and negative correlation (**Supplementary Figures S1C, D**). Through these analyses, we concluded that lower GMR-score and less immune cell infiltration are the key reasons for the development of BC.

### Unraveling GMR complexities at single-cell level

**Figure 3:**
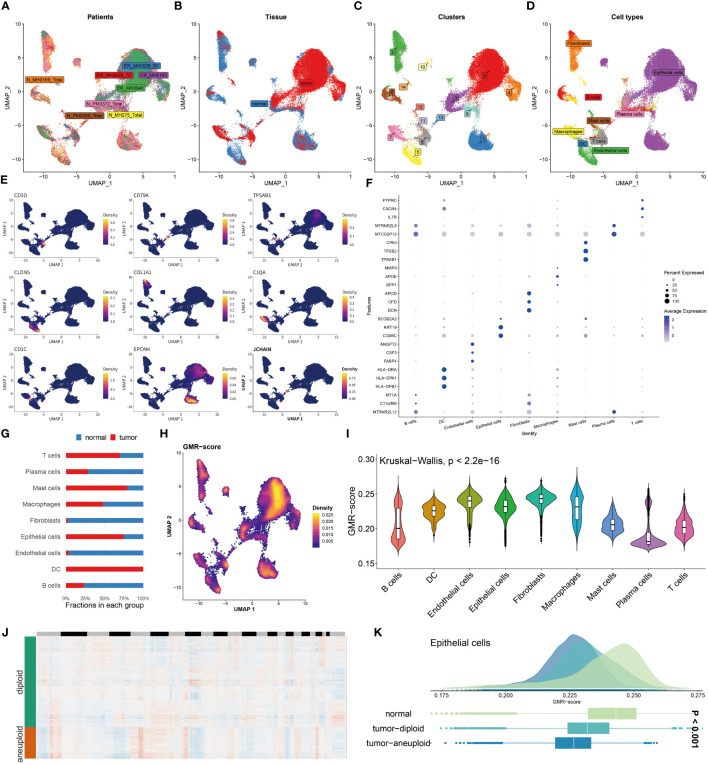
Unraveling GMR complexities at single-cell level. **(A, B)** Distribution of cells collected from tumor and normal tissues of eight patients. **(C, D)** Distribution of cell clusters and annotated cell types. **(E)** UMAP plots showing the expression levels of representative marker genes representing nine cell subtypes. **(F)** Top 3 differentially expressed genes in each cell type. **(G)** A stacked bar chart showing the fractions of each cell type in normal and tumor tissues. **(H)** UMAP plots showing the distribution of GMR-scores in each cell. **(I)** Violin plot demonstrating the difference of GMR-score in each cell type. **(J)** CopyKat algorithm evaluates the genomic variations. **(K)** Comparison of GMR-score among normal, tumor diploid and aneuploid epithelial cells.

To explore the GMR activity among different immune infiltrating cells, we enrolled 8 BC patients including tumor and normal tissues and conducted in-depth exploration of the single-cell data (**Figures 3A, B**). We then divided the 43,766 cells into 15 clusters and ultimately annotated 9 cell types (**Figures 3C, D**). The representative markers and top 3 DEGs for each cell type were demonstrated (**Figures 3E, F**). Among the annotated cell types, we observed the decrease in the Plasma cell, Macrophage, Fibroblast, Endothelial cell and B cell in tumor tissue compared to the normal group, the percentage of T cell, Mast cell, Epithelial cell and DC were significantly increased in tumor tissues (**Figure 3G**). The Ucell algorithm was used to calculate the GMR-scores for each cell (**Figure 3H**). Subsequently, based on the Kruskal-Wallis method, the correlation between GMR-score and nine cell types was estimated (**Figure 3I**). To explore the GMR-score in cancer cell, we performed copyKAT analysis of Epithelial cells (**Figure 3J**). We observed continuous reduction of GMR-score in normal tissue, tumor-diploid and tumor-aneuploid, which were in keeping with the bulk sequence results (**Figure 3K**).

### Illuminating the dynamics of cell-cell interactions in BC

**Figure 4:**
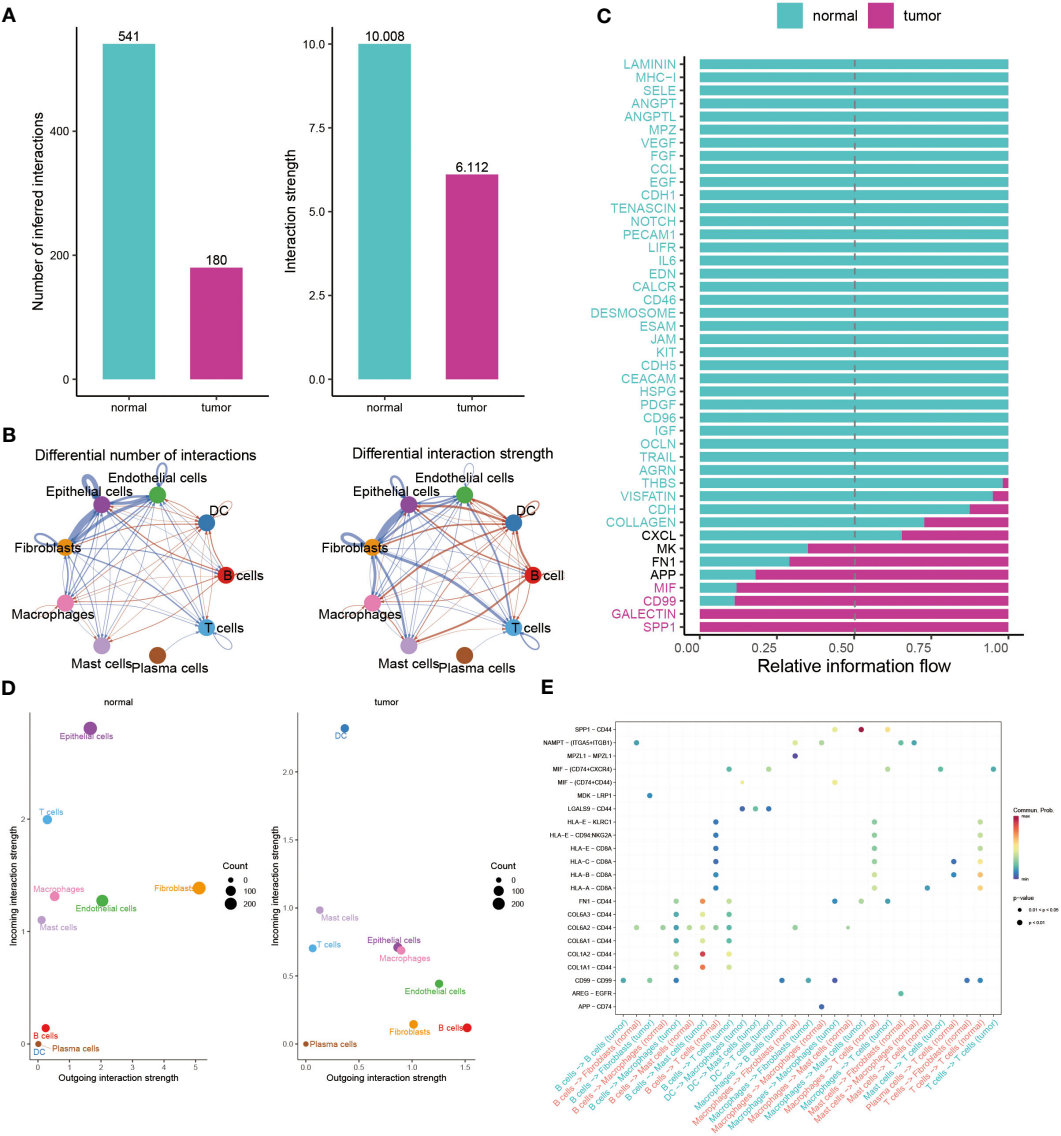
Illuminating the dynamics of cell-cell interactions in BC. **(A, B)** Bar and circle charts showing the differences in the number of interactions (left), strength of interactions (right) in the network of cell-cell communication between normal and tumor groups. **(C)** Stacked plots exhibiting the differences in intercellular signaling pathways between tumor and normal groups. Green and red colors denote up-regulated signaling pathways in normal and tumor samples. **(D)** Scatter plot illustrating the difference in incoming interaction strengths in normal groups (left) and tumor patients (right). Larger circles indicate stronger strengths. **(E)** Dot plot presenting the distribution of distinctive signaling molecules in T cells, B cells, and macrophages between the two groups.

Subsequently, we utilized Cellchat analysis to identify alterations in the quantity and intensity of cellular communication between normal cells and tumor cells. We found that the number and intensity of interactions between BC cells are significantly lower than those of normal cells (**Figure 4A**). In particular, compared with the normal group, the communication of epithelial cells to endothelial cells, Fibroblasts, T cells has been reduced, while the communication between B Cells and epithelial is more frequent (**Figure 4B**). We further explored the specific pathways between the normal and tumor groups by comparing the differences in the interactions. Signal pathways such as SPP1, GALECTIN, CD99, MIF, APP, FN1, and MK are significantly active in BC patients, while LAMININ, MHC-1, and SELE exhibit greater activity in normal individuals (**Figure 4C**). In addition, to continuously detect changes in the submission or acquisition signals between different groups, a comparison based on the intensity of 2D spatial outgoing and incoming interactions is conducted. The scatter plot shows that epithelial cells and T cells serve as the main sources in the normal group, while DC and Mast cells are significant sources in BC patients (**Figure 4D**). Finally, the plot shows a stronger possibility of interaction among immune cells, including B cells, DC, Macrophages, Mast cells, and T cells in the BC group, while the communication between LGALS9 and CD44, MDK and LRP1, and MIF and CD74+CXCR4/CD44 is unique to the BC group (**Figure 4E**).

### Developing the GMR prognosis model through machine learning

**Figure 5:**
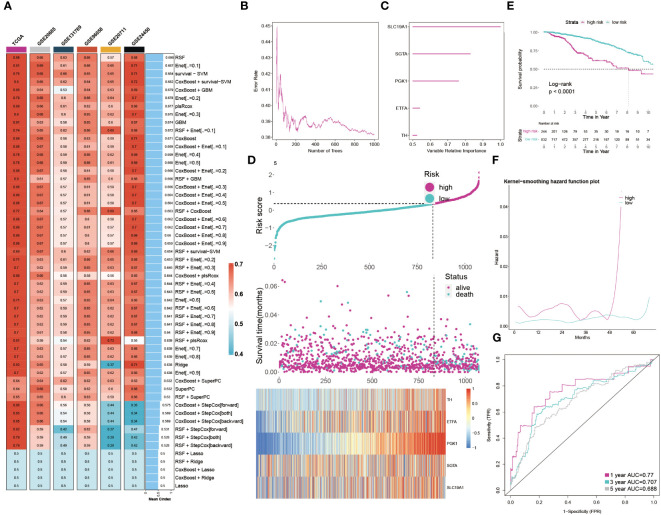
Developing the GMR prognosis model through machine learning. **(A)** The C-index of 54 machine learning algorithm combinations in six cohorts. **(B)** The error rate in several different trees. **(C)** The importance of each GMR gene. **(D)** Distribution between risk score and survival status and gene expression. **(E)** KM survival illustrates the survival probability in these two groups. **(F)** The kernel-smoothing hazard function plot demonstrates the correlation between relapse hazard and moths in two populations. **(G)** The ROC curves visualize the AUC values of the GMR-model at one-, three-, and five-year.

The results clearly indicate a significant link between GMR related genes and BC. We then employed machine learning analyses on the TCGA training group and five external cohorts, using 54 combinations of 10 machine learning methods. This approach yielded an average C-index for the six groups (**Figure 5A**). Our analysis led to the selection of Random Survival Forest for constructing a GMR prognosis model, pinpointing five key genes (SLC19A1, SGTA, PGK1, ETFA, TH) linked to BC prognosis (**Figures 5B, C**). Patients were then stratified into high-risk and low-risk groups based on risk scores derived from these genes. The heatmap showed these genes were notably upregulated in the high-risk group (**Figure 5D**). Kaplan-Meier survival curves revealed significantly higher survival rates in the low-risk group (**Figure 5E**). Recurrence risk predictions indicated a considerably increased risk in the high-risk group (**Figure 5F**). The ROC curve, assessing the prognostic model’s efficiency (**Figure 5G**), demonstrated AUC values above 0.5 across the first, third, and fifth years, affirming the model’s predictive accuracy and reliability. Considering the dataset’s correlation between risk scores and survival status, we concluded that the low-risk group has a better survival outlook, whereas the high-risk group faces a higher probability of mortality.

### Assessment and validation of the GMR-model

**Figure 6:**
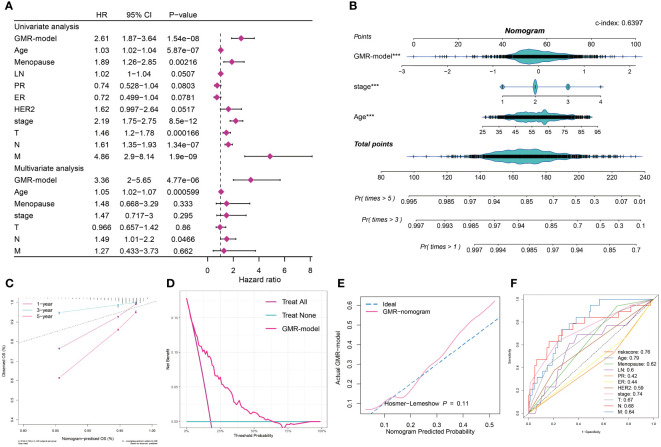
Assessment and validation of the GMR-model. **(A)** Univariate and multivariate Cox regression analysis of prognostic ability for GMR-model and other clinical pathological features. **(B)** GMR-nomogram was built consisting of risk score, age and stage index to predict 1-, 3-, and 5-year OS of BC. **(C)** Correction curve demonstrating the observed OS (%) and the predicted OS (%) of the nomogram. **(D)** DCA curves indicates two extreme lines drawn from treat all and treat none, respectively. **(E)** Evaluate the accuracy of GMR column charts and ideal curves using the Hosmer-Lemeshow method. **(F)** 11 ROC curves respectively unfolding the corresponding AUC values of the risk score and ten clinicopathological indexes. ***P < 0.001.

In this study, univariate and multivariate Cox regression analyses were employed to assess the independence of our prognostic model relative to other clinical factors in BC patients. Univariate analysis revealed that several indicators, such as risk score, menopause status, stage, and TNM classification, significantly impacted survival rates. Notably, multivariate analysis showed that both risk score and age met the significance threshold (P < 0.05), affirming the predictive independence of our GMR-model for BC patient outcomes (**Figure 6A**). Recognizing the clinical importance of staging, we developed a GMR-nomogram that integrates risk score, stage, and age to accurately forecast one-, three-, and five-year survival probabilities for BC patients (**Figure 6B**). The calibration curve is used to calibrate the accuracy of the 1-year, 3-year, and 5-year nomograms, indicating a high degree of consistency with actual survival rates (**Figure 6C**). In addition, the GMR-model chart is higher than the two extreme curves (Treat All and Treat None), indicating that the GMR-column chart has reliable predictive ability (**Figure 6D**). Furthermore, there was no statistically significant difference (p > 0.05) between the predicted values of the GMR column chart and the ideal observed values, further demonstrating the predictive ability of the GMR model (**Figure 6E**). We also compared our model with clinical pathological factors and found that the GMR-model better reflects the prognostic correlation of BC with other pathological factors besides age (**Figure 6F**). Then, we compared the predictive ability of the GMR-model and these 100 signatures in the training group and 10 external cohorts using the C-index. Our GMR-model showed significantly better superior accuracy than other models in almost all queues (ranking first in five queues, second in five queues, and fourth in one queue), revealing the robustness of the GMR-model (**Supplementary Figure S2**).

### Multi-omics analysis of genetic variations

**Figure 7:**
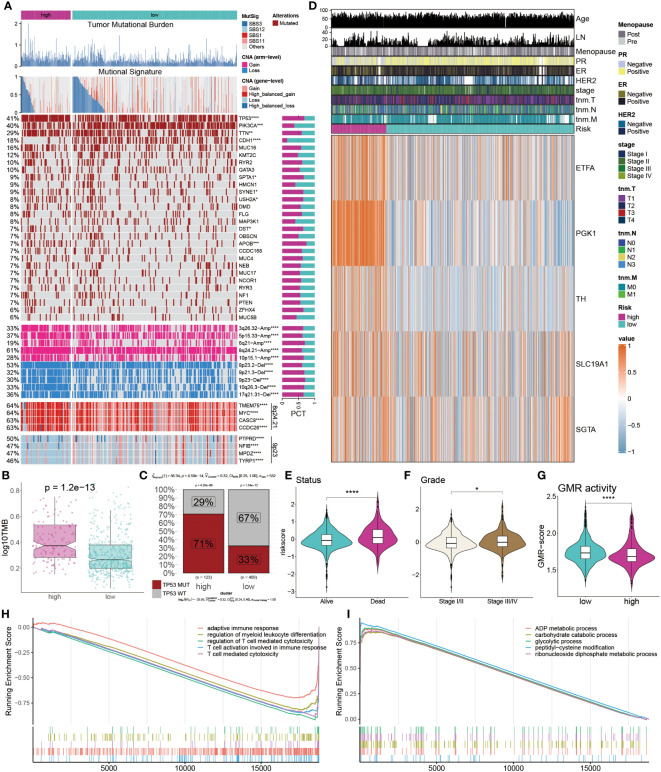
Multi-omics analysis of genetic variations. **(A)** Distribution of TMB, mutational signatures, gene mutation, CNVs and oncogenes. **(B)** Comparison of TMB between the two groups. **(C)** Proportions of TP53 mutation in the two groups. **(D)** Heatmap displays the distribution of GMR regulators and clinicopathological factors in the two groups. **(E, F)** Comparison of survival status and clinical grade between GMR groups. **(G, H)** Functional annotation and gene enrichment analysis for high-risk patients. *P < 0.05, ***P < 0.001, ****P < 0.0001.

To systematically evaluate genomic heterogeneity based on the GMR-model, we calculated gene mutations and CNV between high-risk and low-risk groups. We observed the significant mutation frequency changes of TP53, PIK3CA, TTN, CDH1 and APOB (P < 0.05). Further analysis revealed that amplification or deletion of copy numbers were also detected in high-risk BC patients, such as 3p26.32, 5p15.33, 6q21, 8q24.21, and 10p15.1 amplification and 8p23, 9p21.3, 9p23, 10q26.3, and 17q21.31 deletion. In addition, our findings were confirmed by the amplification of oncogenes TMEM75, MYC, CASC8, CCDC26 in the 8q24.21 fragment and the deletion of tumor suppressor genes PYPRD, NFIB, MPZD, and TYRP1 in the 9p23 fragment (**Figure 7A**).

TMB was notably higher in the high-risk group compared to the low-risk group (**Figure 7B**). Further examination of the key tumor suppressor gene TP53 revealed a significantly elevated mutation rate in the high-risk group (**Figure 7C**). Additionally, a heatmap illustrated the expression profiles of eight GMR modulators, with ETFA, PGK1, TH, SLC19A1, and SGTA exhibiting higher expression in the high-risk group (**Figure 7D**). Risk assessment considering survival status and clinical staging in BC patients indicated that those with poorer survival outcomes and less favorable clinical stages had higher risk scores (**Figures 7E, F**). We further observed the GMR-score were markedly lower in the high-risk group compared to low-risk cohorts (**Figure 7G**). To understand potential BC mechanisms, functional annotation and gene enrichment analyses were performed. The results suggested that, in the high-risk group compared to the low-risk group, immune-related processes were suppressed while metabolic pathways were activated (**Figures 7H, I**). These findings provide insights into the mechanisms potentially driving BC progression.

### Immune landscape diversity across GMR groups

**Figure 8:**
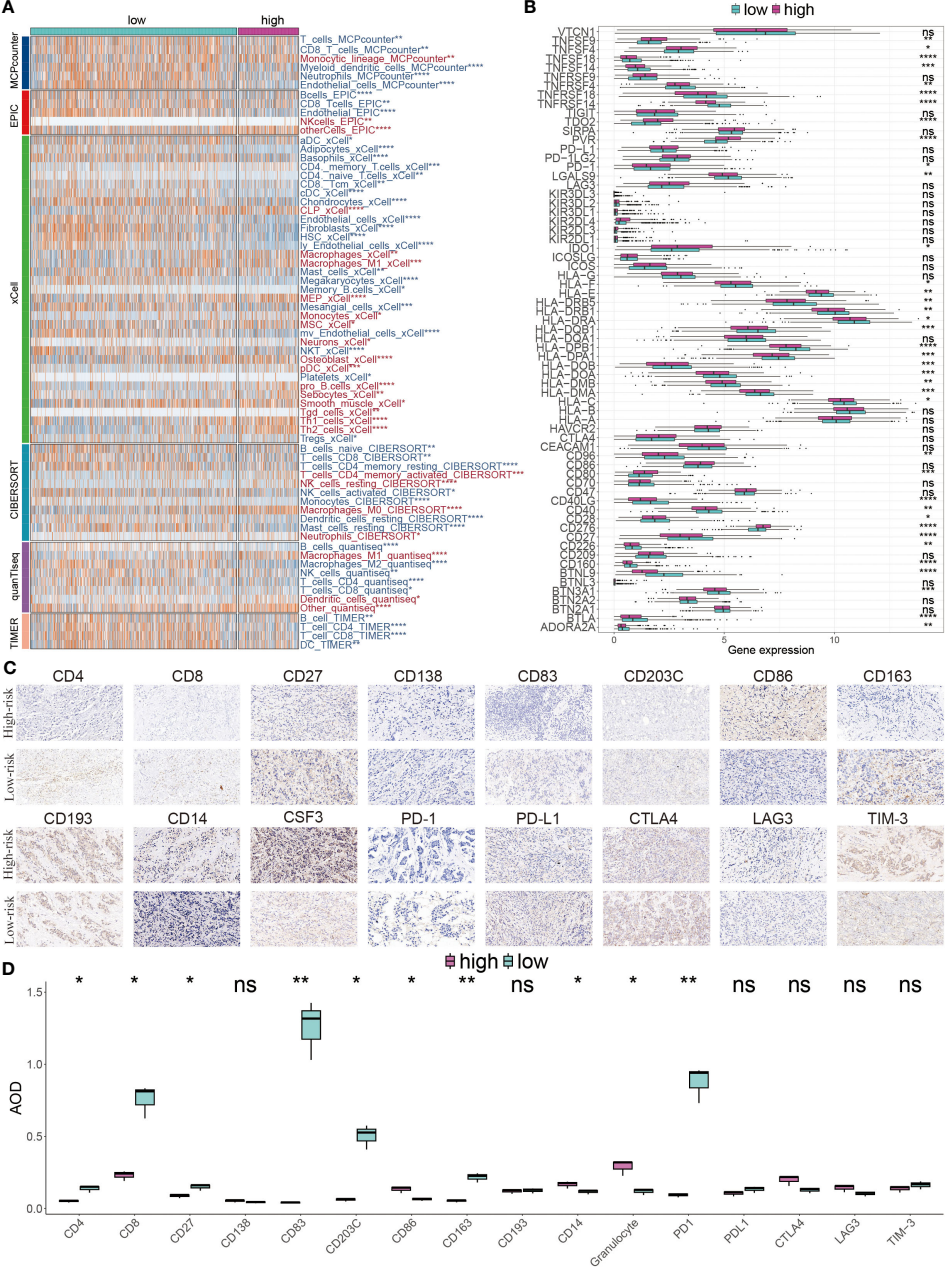
Immune landscape diversity across GMR groups. **(A)** The distribution of distinctive infiltrated immune cells between two risk subgroups calculated using multiple algorithms. **(B)** The differential expression profiles of immune-checkpoint genes between two GMR groups. **(C)** IHC image of infiltrated immune cells targeting the reproductive makers. **(D)** Statistical result of **(C)**. *P < 0.05, **P < 0.01, ***P < 0.001, ****P < 0.0001.

We next explored the difference of tumor infiltrating lymphocytes (TILs) between the two subgroups. Six immune infiltrating algorithms were used to estimate different TIL. In low-risk patients, the tumor microenvironment is characterized by a substantial infiltration of immune cells, including CD4 T cells and CD8 T cells, which are classified as tumor-infiltrating lymphocytes (TILs), as well as a notable presence of M2 macrophages and DCs. In the high-risk group, the relative content of M1 macrophages, pro-B cells, Th1 cells, and Th2 cells were significantly increased (**Figure 8A**). We further showed that patients with low risk exhibited increased immune cell infiltration, marked by a greater prevalence of immune-checkpoint genes, correlating with better prognoses (**Figure 8B**). For a more in-depth examination of the TME and to confirm our findings, IHC staining, targeting key cellular markers and immune-checkpoint genes, was conducted. The representative IHC images and statistical outcomes are presented in **Figures 8C, D**.

### Predicting immunotherapy response using the GMR-model

**Figure 9:**
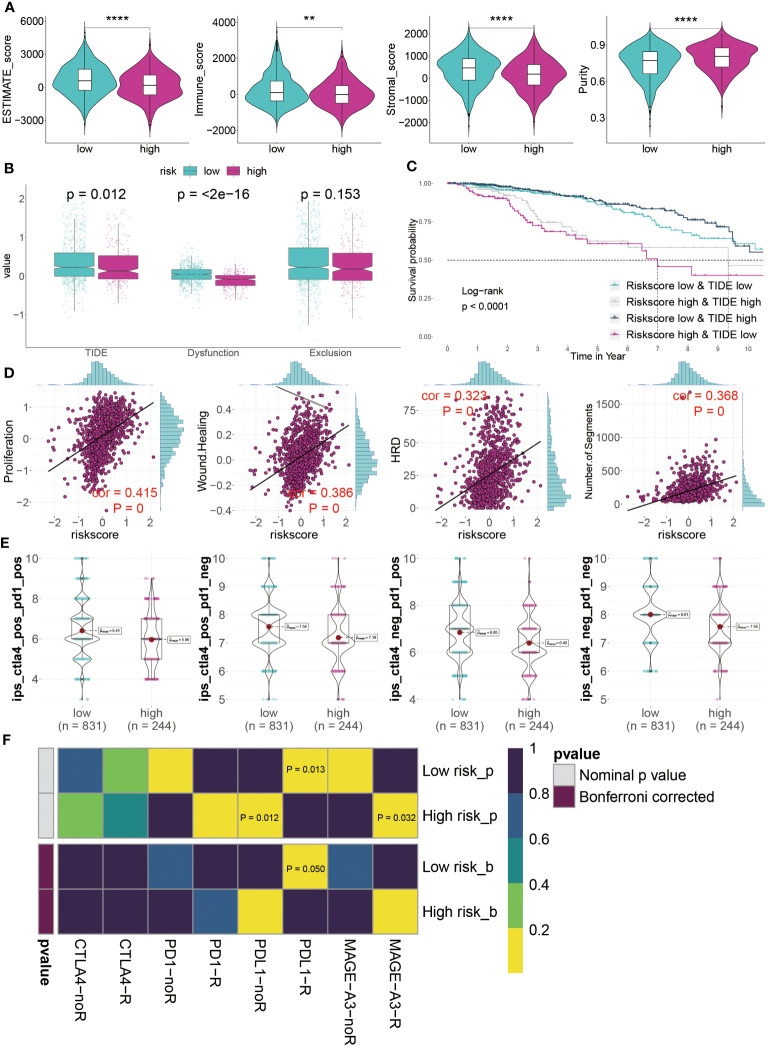
Predicting immunotherapy response using the GMR-model. **(A)** Distinctive scores of estimate algorithm, including estimate scores, immune scores, stromal scores, and tumor purity. **(B)** Comparison of TIDE algorithm between GMR groups. **(C)** KM survival curve analysis of patients with different combinations of risk scores and TIDE in TCGA cohort. **(D)** The relationship between proposition, wound healing, homologous recognition defect, and number of segments risk scores. **(E)** IPS (Immunophenoscore) value of each combination among two risk groups. **(F)** Putative ICIs therapy response in two risk BC patients. **P < 0.01, ****P < 0.0001.

Our analysis of the TME suggested that those at low risk might respond better to immunotherapy. This hypothesis stemmed from their higher levels of immune cell infiltration and increased expression of ICI genes. To validate this, we utilized ESTIMATE analysis, which showed significantly higher ESTIMATE, immune, and stromal scores, alongside lower tumor purity, in the low-risk group (**Figure 9A**). TIDE analysis, a tool for predicting immunotherapy efficacy, generally correlates negatively with treatment response. Our findings indicated higher TIDE and Dysfunction values in low-risk patients, although differences in Exclusion scores between low- and high-risk groups were not statistically significant (P > 0.05) (**Figure 9B**). KM survival curves, representing four different risk and TIDE combinations, suggested that low-risk patients with high TIDE values had better outcomes, underscoring the pivotal role of risk score in prognosis (**Figure 9C**).

Furthermore, we assessed tumor immunogenicity based on proliferation, wound healing, homologous recombination deficiency, and chromosomal segments. Our results showed a positive correlation between these indices and risk score, indicating poorer prognoses in high-risk BC patients (**Figure 9D**). The suitability of different patient groups for ICI therapy remains unclear. However, immune profiling score (IPS) analysis using the TCGA dataset revealed exceptionally high scores in low-risk BC patients, suggesting a greater likelihood of benefiting from immunotherapy, whether standalone or in combination (**Figure 9E**). Finally, evaluating responses to PD1, PDL1, CTLA4, and MAGE-A3 treatments indicated that low-risk patients primarily respond to anti-PD-L1 therapy (Bonferroni corrected P < 0.05) (**Figure 9F**). In summary, our GMR-model effectively predicts ICI therapy responsiveness across different groups, with low-risk BC patients seemingly more favorable candidates for this treatment approach in a clinical setting.

### Chemotherapy response and new treatment avenues for high-risk BC

**Figure 10:**
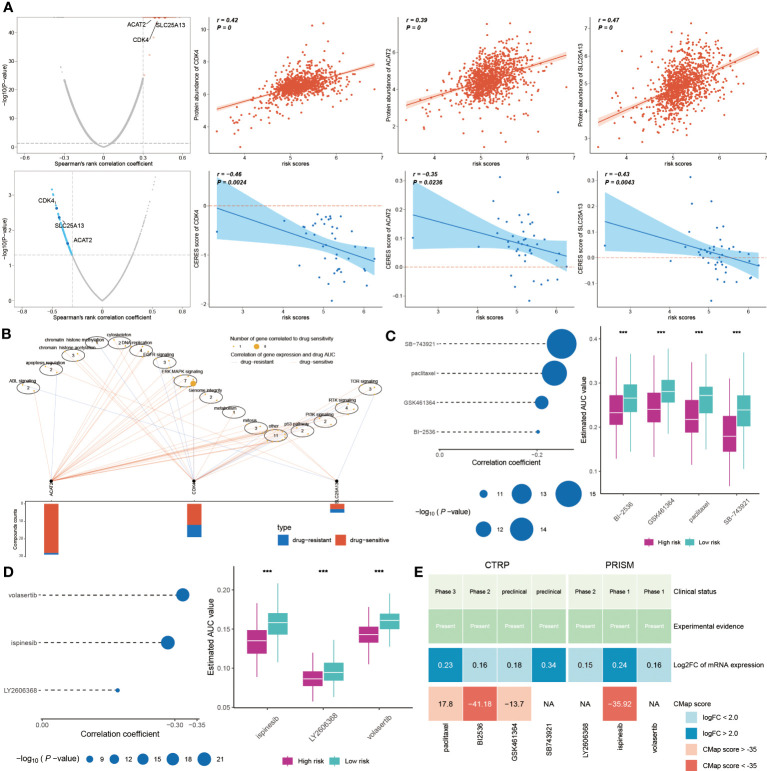
Chemotherapy response and new treatment avenues for high-risk BC. **(A)** Spearman correlations between risk score and gene abundance and CERES score of drug targets. **(B)** Spearman correlation between gene expression of potential targets and drug sensitivity across cancer cell line. **(C, D)** Four compounds obtained from CTRP and three compounds obtained from PRISM were subjected to Spearman correlation analysis. The block diagram correspondingly shows the difference in estimated AUC values of different compounds within the two groups. **(E)** This graph displays the clinical status, experimental evidence, mRNA expression levels, and CMap scores of four drugs for CTRP and three drugs for PRISM, respectively. ***P < 0.001.

In exploring new cancer treatments, targeted therapy has been considered, yet chemotherapy remains vital in clinical cancer management. Our model, aimed at predicting chemotherapy responses in BC patients, is particularly crucial for those at high risk. A key step is identifying therapeutic targets to tackle currently undruggable challenges. Using Spearman’s correlation analysis, we discovered four proteins more abundant in high-risk patients, suggesting a greater susceptibility to chemotherapy in this group. The CERES scores corroborate these proteins as therapeutic targets for this demographic (**Figure 10A**). Additionally, these proteins associated anti-cancer drugs demonstrated increased drug sensitivity (**Figure 10B**). Consequently, CDK4, SLC25A13, and ACAT2 are proposed as potential therapeutic targets for high-risk BC.

Further, we sought potential drugs using PRISM and CTRP datasets. Seven candidate compounds were identified: paclitaxel, BI-2536, GSK461364, and SB-743921 from CTRP (**Figure 10C**), and LY2606368, ispinesib, and volasertib from PRISM (**Figure 10D**). Lower AUC values in high-risk patients indicated better chemotherapy response. However, to determine the most effective drugs, we conducted a multi-faceted analysis, incorporating clinical status, experimental evidence, mRNA expression levels, and CMap scores. Based on a CMap score criterion of < -35, BI-2536 and ispinesib emerged as the chosen therapeutic drugs for high-risk BC (**Figure 10E**, **Supplementary Table S3**).

## Discussion

Glutamine metabolism is one of the key metabolic pathways in all cells, and normal glutamine metabolism is particularly important for the normal occurrence and development of cells. Thus, abnormal glutamine metabolism often brings irreversible damage to cells, which is considered as the key factor to promote the progress of solid tumors such as BC (9). Therefore, based on the particularity of glutamine metabolism and the status of treatment and prognosis of BC, we urgently need to identify reliable and accurate glutamine metabolism related markers to predict the survival and immune response of BC patients. In this project, we obtained regulatory factors related to GMR and analyzed the relationship between GMR and BC heterogeneity.

The relevance of scoring systems in evaluating the activities of metabolic pathways, including glutamine metabolism, in cancer has been well-documented in the literature. For instance, the study by Giunchi et al. reviews the metabolic landscape of prostate cancer, emphasizing metabolic alterations and their potential as diagnostic and therapeutic targets, underscoring the importance of metabolic pathways in cancer development and progression (36). Similarly, Sudarshan et al. discuss the metabolic effects of genes and signaling pathways implicated in renal cancer, highlighting the role of altered metabolism in the pathogenesis of this malignancy (37). This further validates the use of scoring systems like our GMR-score to quantitatively assess metabolic alterations in cancer. These studies collectively support the premise that metabolic scoring systems can effectively represent the activity levels of specific metabolic pathways, including glutamine metabolism, and their association with cancer. By adopting a quantitative approach to assess these metabolic activities, our GMR-score provides a robust tool for understanding the metabolic reprogramming in cancer, reinforcing the validity of our approach in linking specific metabolic pathways with cancer-associated gene expression changes.

While highlighting the role of GMR in the prognosis of BC, we constructed a model of 5 GMR related genes based on one training set and five external datasets and compared it with 100 existing models. The results indicate that the GMR prediction model is more robust, independent, and reliable. Subsequently, GMR-nomogram was established to predict the survival probability of BC patients at 1, 3, and 5 years, which has the best predictive ability compared to other clinical pathological features based on AUC values. Of note, five GMR genes (SLC19A1, SGTA, PGK1, ETFA, TH) were upregulated in the high-risk group.

Our discussion extended to the genetic differences between the two risk groups. The low-risk group was characterized by low TMB and fewer mutated genes, whereas the high-risk group exhibited higher TMB and more mutations in genes such as TP53 and PIK3CA. TP53, a quintessential tumor suppressor gene, has been implicated in BC progression and poor prognosis due to its mutations (38). CNV analysis revealed that high-risk patients also displayed notable aberrations in gene amplifications and deletions. The human genome locus 8q24.21, often referred to as a “gene desert” due to its sparse protein-coding genes, has been linked to various cancer phenotypes despite covering a 4.1MB region (39). This area houses the well-studied oncogene MYC, implicated in approximately 20% of human cancers (40). Also located in this region is the OCT4 pseudogene POU5F1B, which has been observed to amplify in cancers (41). Beyond oncogenic protein-coding genes, the 8q24.21 region is a hub for many lncRNAs associated with different cancers. These lncRNAs function independently of MYC, highlighting the complexity and significance of this genomic region in cancer biology and risk stratification (42). This indicates that due to the role of MYC in accelerating tumor progression, it further promotes tumor development. According to reports, 9p23 deficiency may lead to chronic myeloid leukemia (43). CDKN2A and CDKN2B are tumor suppressor genes located at 9p21, and their deletion inevitably increases tumor risk. These characteristics indicate poor prognosis in BC patients, but also lay the foundation for the response of low-risk patients to ICI treatment.

Our findings illustrate that the GMR-model effectively categorizes tumors into low-risk and high-risk groups, which correspond to distinct immune phenotypes within the TME. Specifically, the low-risk group demonstrated a high infiltration of CD8^+^ T cells, aligning with an “immune-inflamed” phenotype, indicative of a robust antitumor immune response. Conversely, the high-risk group was characterized by an “immune desert” phenotype, with a notable presence of TP53 mutations, suggesting a reduced capacity for eliciting an effective immune response. The distinction between “immune-inflamed” and “immune desert” phenotypes is crucial for predicting the efficacy of immunotherapeutic interventions. Traditionally, the direct prediction of immunotherapy responses from genome-wide gene expression data has been challenging. However, our GMR-model provides a nuanced framework that not only encapsulates the interplay between genomic alterations and immune responses but also enhances the predictive capabilities for immunotherapy outcomes beyond the binary classification of risk levels. This underscores the importance of considering both genomic and immune contexts in designing personalized immunotherapeutic strategies.

BI 2536 is a highly selective and potent PLK 1 inhibitor that can regulate the malignant behavior of gastric cancer cells in combination with cisplatin (44). Schöffski P. et al. conducted parallel phase II experiments on the treatment of BC with BI 2536 over a decade ago (45). Ispinesib (also known as SB-715992, CK-0238273) was the first small molecule inhibitor of KSP, an effective, specific, and allosteric inhibitor. Inhibiting KSP activity by preventing the release of ADP without altering the release of KSP-ADP complex in microtubules. It is reported that ispinesib inhibits cell proliferation by inducing medium-term cell division dysfunction of pancreatic cancer cells (46). These results enhance the persuasiveness of these two drugs for treating high-risk BC patients.

## Conclusion

Our study introduces a groundbreaking GMR prognostic model for breast cancer, offering precise prognosis and treatment response predictions. This model distinguishes high-risk patients, guiding targeted chemotherapy, and identifies low-risk patients likely to benefit from immunotherapies. Highlighting the pivotal role of glutamine metabolism-related genes in breast cancer, the GMR model marks a significant step in personalized cancer therapy, promising enhanced patient care and treatment outcomes.

## Data availability statement

The datasets presented in this study can be found in online repositories. The names of the repository/repositories and accession number(s) can be found in the article/**Supplementary Material**.

## Ethics statement

The studies involving humans were approved by Ethics Committee of Guizhou Provincial People’s Hospital. The studies were conducted in accordance with the local legislation and institutional requirements. The participants provided their written informed consent to participate in this study.

## Author contributions

XL (1^st^ author): Data curation, Formal analysis, Investigation, Validation, Writing – original draft. XL (2^nd^ author): Data curation, Formal analysis, Investigation, Methodology, Writing – original draft. BY: Investigation, Resources, Supervision, Validation, Writing – review & editing. SS: Methodology, Software, Supervision, Validation, Writing – review & editing. SW: Resources, Validation, Writing – review & editing. FY: Conceptualization, Funding acquisition, Resources, Supervision, Validation, Writing – review & editing. TW: Conceptualization, Data curation, Formal analysis, Funding acquisition, Investigation, Methodology, Validation, Visualization, Writing – original draft, Writing – review & editing.

## References

[1] Britt KL Cuzick J Phillips. KA. Key steps for effective breast cancer prevention. Nat Rev Cancer. (2020) 20:417–36. doi: 10.1038/s41568-020-0266-x 32528185

[2] Wilkinson L Gathani T. Understanding breast cancer as a global health concern. Br J Radiol. (2022) 95:20211033. doi: 10.1259/bjr.20211033 34905391 PMC8822551

[3] Sun YS Zhao Z Yang ZN Xu F Lu HJ Zhu ZY. Risk factors and preventions of breast cancer. Int J Biol Sci. (2017) 13:1387–97. doi: 10.7150/ijbs.21635 PMC5715522 29209143

[4] Wang S Xiong Y Zhang Q Su D Yu C Cao Y. Clinical significance and immunogenomic landscape analyses of the immune cell signature based prognostic model for patients with breast cancer. Brief Bioinform. (2021) 22:bbaa311. doi: 10.1093/bib/bbaa311 33302293

[5] DeBerardinis RJ Lum JJ Hatzivassiliou G Thompson. CB. The biology of cancer: metabolic reprogramming fuels cell growth and proliferation. Cell Metab. (2008) 7:11–20. doi: 10.1016/j.cmet.2007.10.002 18177721

[6] Tao H Zhong X Zeng A Song. L. Unveiling the veil of lactate in tumor-associated macrophages: a successful strategy for immunometabolic therapy. Front Immunol. (2023) 14:1208870. doi: 10.3389/fimmu.2023.1208870 37564659 PMC10411982

[7] Ma G Zhang Z Li P Zhang Z Zeng M Liang Z. Reprogramming of glutamine metabolism and its impact on immune response in the tumor microenvironment. Cell Commun Signal. (2022) 20:114. doi: 10.1186/s12964-022-00909-0 35897036 PMC9327201

[8] Carrascosa JM Martínez P Núñez de Castro. I. Nitrogen movement between host and tumor in mice inoculated with Ehrlich ascitic tumor cells. Cancer Res. (1984) 44:3831–5. 6146402

[9] Wang L Xu T Yang X Liang Z Zhang J Li D. Immunosuppression induced by glutamine deprivation occurs *via* activating PD-L1 transcription in bladder cancer. Front Mol Biosci. (2021) 8:687305. doi: 10.3389/fmolb.2021.687305 34805266 PMC8602840

[10] Zhang J Pavlova NN Thompson. CB. Cancer cell metabolism: the essential role of the nonessential amino acid, glutamine. EMBO J. (2017) 36:1302–15. doi: 10.15252/embj.201696151 PMC5430235 28420743

[11] Pal B Chen Y Vaillant F Capaldo BD Joyce R Song X. A single-cell RNA expression atlas of normal, preneoplastic and tumorigenic states in the human breast. EMBO J. (2021) 40:e107333. doi: 10.15252/embj.2020107333 33950524 PMC8167363

[12] McGinnis CS Murrow LM Gartner. ZJ. DoubletFinder: doublet detection in single-cell RNA sequencing data using artificial nearest neighbors. Cell Syst. (2019) 8:329–337.e4. doi: 10.1016/j.cels.2019.03.003 30954475 PMC6853612

[13] Domínguez Conde C Xu C Jarvis LB Rainbow DB Wells SB Gomes T. Cross-tissue immune cell analysis reveals tissue-specific features in humans. Science. (2022) 376:eabl5197. doi: 10.1126/science.abl5197 35549406 PMC7612735

[14] Gao R Bai S Henderson YC Lin Y Schalck A Yan Y. Delineating copy number and clonal substructure in human tumors from single-cell transcriptomes. Nat Biotechnol. (2021) 39:599–608. doi: 10.1038/s41587-020-00795-2 33462507 PMC8122019

[15] Jin S Guerrero-Juarez CF Zhang L Chang I Ramos R Kuan CH. Inference and analysis of cell-cell communication using CellChat. Nat Commun. (2021) 12:1088. doi: 10.1038/s41467-021-21246-9 33597522 PMC7889871

[16] Ashburner M Ball CA Blake JA Botstein D Butler H Cherry JM. Gene ontology: tool for the unification of biology. Gene Ontology Consortium Nat Genet. (2000) 25:25–9. doi: 10.1038/75556 PMC3037419 10802651

[17] Kanehisa M Sato Y Kawashima M Furumichi M Tanabe. M. KEGG as a reference resource for gene and protein annotation. Nucleic Acids Res. (2016) 44:D457–62. doi: 10.1093/nar/gkv1070 PMC4702792 26476454

[18] Yu G Wang LG Han Y He. QY. clusterProfiler: an R package for comparing biological themes among gene clusters. OMICS. (2012) 16:284–7. doi: 10.1089/omi.2011.0118 PMC3339379 22455463

[19] Hänzelmann S Castelo R Guinney. J. GSVA: gene set variation analysis for microarray and RNA-seq data. BMC Bioinf. (2013) 14:7. doi: 10.1186/1471-2105-14-7 PMC3618321 23323831

[20] Andreatta M Carmona SJ. UCell: Robust and scalable single-cell gene signature scoring. Comput Struct Biotechnol J. (2021) 19:3796–8. doi: 10.1016/j.csbj.2021.06.043 PMC8271111 34285779

[21] Liu Z Guo C Dang Q Wang L Liu L Weng S. Integrative analysis from multi-center studies identities a consensus machine learning-derived lncRNA signature for stage II/III colorectal cancer. EBioMedicine. (2022) 75:103750. doi: 10.1016/j.ebiom.2021.103750 34922323 PMC8686027

[22] Wang L Liu Z Liang R Wang W Zhu R Li J. Comprehensive machine-learning survival framework develops a consensus model in large-scale multicenter cohorts for pancreatic cancer. Elife. (2022) 11:e80150. doi: 10.7554/eLife.80150 36282174 PMC9596158

[23] Becht E Giraldo NA Lacroix L Buttard B Elarouci N Petitprez F. Estimating the population abundance of tissue-infiltrating immune and stromal cell populations using gene expression. Genome Biol. (2016) 17:218. doi: 10.1186/s13059-016-1070-5 27765066 PMC5073889

[24] Racle J Gfeller D. EPIC: A tool to estimate the proportions of different cell types from bulk gene expression data. Methods Mol Biol. (2020) 2120:233–48. doi: 10.1007/978-1-0716-0327-7_17 32124324

[25] Aran D Hu Z Butte. AJ. xCell: digitally portraying the tissue cellular heterogeneity landscape. Genome Biol. (2017) 18:220. doi: 10.1186/s13059-017-1349-1 29141660 PMC5688663

[26] Newman AM Liu CL Green MR Gentles AJ Feng W Xu Y. Robust enumeration of cell subsets from tissue expression profiles. Nat Methods. (2015) 12:453–7. doi: 10.1038/nmeth.3337 PMC4739640 25822800

[27] Finotello F Mayer C Plattner C Laschober G Rieder D Hackl H. Molecular and pharmacological modulators of the tumor immune contexture revealed by deconvolution of RNA-seq data. Genome Med. (2019) 11:34. doi: 10.1186/s13073-019-0638-6 31126321 PMC6534875

[28] Li T Fan J Wang B Traugh N Chen Q Liu JS. TIMER: A web server for comprehensive analysis of tumor-infiltrating immune cells. Cancer Res. (2017) 77:e108–10. doi: 10.1158/0008-5472.Can-17-0307 PMC6042652 29092952

[29] Zeng D Ye Z Shen R Yu G Wu J Xiong Y. IOBR: multi-omics immuno-oncology biological research to decode tumor microenvironment and signatures. Front Immunol. (2021) 12:687975. doi: 10.3389/fimmu.2021.687975 34276676 PMC8283787

[30] Yoshihara K Shahmoradgoli M Martinez E Vegesna R Kim H Torres-Garcia W. Inferring tumour purity and stromal and immune cell admixture from expression data. Nat Commun. (2013) 4:2612. doi: 10.1038/ncomms3612 24113773 PMC3826632

[31] Jiang P Gu S Pan D Fu J Sahu A Hu X. Signatures of T cell dysfunction and exclusion predict cancer immunotherapy response. Nat Med. (2018) 24:1550–8. doi: 10.1038/s41591-018-0136-1 PMC6487502 30127393

[32] Meyers RM Bryan JG McFarland JM Weir BA Sizemore AE Xu H. Computational correction of copy number effect improves specificity of CRISPR-Cas9 essentiality screens in cancer cells. Nat Genet. (2017) 49:1779–84. doi: 10.1038/ng.3984 PMC5709193 29083409

[33] Yang C Huang X Li Y Chen J Lv Y Dai. S. Prognosis and personalized treatment prediction in TP53-mutant hepatocellular carcinoma: an in silico strategy towards precision oncology. Brief Bioinform. (2021) 22:bbaa164. doi: 10.1093/bib/bbaa164 32789496

[34] Wang T Li T Li B Zhao J Li Z Sun M. Immunogenomic landscape in breast cancer reveals immunotherapeutically relevant gene signatures. Front Immunol. (2022) 13:805184. doi: 10.3389/fimmu.2022.805184 35154121 PMC8829007

[35] Wang T Ba X Zhang X Zhang N Wang G Bai B. Nuclear import of PTPN18 inhibits breast cancer metastasis mediated by MVP and importin β2. Cell Death Disease. (2022) 13:720. doi: 10.1038/s41419-022-05167-z 35982039 PMC9388692

[36] Giunchi F Fiorentino M Loda. M. The metabolic landscape of prostate cancer. Eur Urol Oncol. (2019) 2:28–36. doi: 10.1016/j.euo.2018.06.010 30929843

[37] Sudarshan S Karam JA Brugarolas J Thompson RH Uzzo R Rini B. Metabolism of kidney cancer: from the lab to clinical practice. Eur Urol. (2013) 63:244–51. doi: 10.1016/j.eururo.2012.09.054 PMC3709870 23063455

[38] Blondeaux E Arecco L Punie K Graffeo R Toss A De Angelis C. Germline TP53 pathogenic variants and breast cancer: A narrative review. Cancer Treat Rev. (2023) 114:102522. doi: 10.1016/j.ctrv.2023.102522 36739824

[39] Huppi K Pitt JJ Wahlberg BM Caplen. NJ. The 8q24 gene desert: an oasis of non-coding transcriptional activity. Front Genet. (2012) 3:69. doi: 10.3389/fgene.2012.00069 22558003 PMC3339310

[40] Dang CV O'Donnell KA Zeller KI Nguyen T Osthus RC Li. F. The c-Myc target gene network. Semin Cancer Biol. (2006) 16:253–64. doi: 10.1016/j.semcancer.2006.07.014 16904903

[41] Hayashi H Arao T Togashi Y Kato H Fujita Y De Velasco MA. The OCT4 pseudogene POU5F1B is amplified and promotes an aggressive phenotype in gastric cancer. Oncogene. (2015) 34:199–208. doi: 10.1038/onc.2013.547 24362523

[42] Wilson C Kanhere A. 8q24.21 locus: A paradigm to link non-coding RNAs, genome polymorphisms and cancer. Int J Mol Sci. (2021) 22:1094. doi: 10.3390/ijms22031094 33499210 PMC7865353

[43] Wafa A Asa'ad M Ikhtiar A Liehr T Al-Achkar. W. Deletion 9p23 to 9p11.1 as sole additional abnormality in a Philadelphia positive chronic myeloid leukemia in blast crisis: a rare event. Mol Cytogenet. (2015) 8:59. doi: 10.1186/s13039-015-0165-0 26244056 PMC4523925

[44] Lian G Li L Shi Y Jing C Liu J Guo X. BI2536, a potent and selective inhibitor of polo-like kinase 1, in combination with cisplatin exerts synergistic effects on gastric cancer cells. Int J Oncol. (2018) 52:804–14. doi: 10.3892/ijo.2018.4255 PMC5807034 29393385

[45] Schöffski P Blay JY De Greve J Brain E Machiels JP Soria JC. Multicentric parallel phase II trial of the polo-like kinase 1 inhibitor BI 2536 in patients with advanced head and neck cancer, breast cancer, ovarian cancer, soft tissue sarcoma and melanoma. The first protocol of the European Organization for Research and Treatment of Cancer (EORTC) Network Of Core Institutes (NOCI). Eur J Cancer. (2010) 46:2206–15. doi: 10.1016/j.ejca.2010.03.039 20471824

[46] Murase Y Ono H Ogawa K Yoshioka R Ishikawa Y Ueda H. Inhibitor library screening identifies ispinesib as a new potential chemotherapeutic agent for pancreatic cancers. Cancer Sci. (2021) 112:4641–54. doi: 10.1111/cas.15134 PMC8586681 34510663

