# Supplementary material for: Enhancing breast cancer outcomes with machine learning-driven glutamine metabolic reprogramming signature
Source: Front Immunol. 2024 May 1;15:1369289. doi: 10.3389/fimmu.2024.1369289 (PMC11097668; doi:10.3389/fimmu.2024.1369289)
Supplement: Supplementary file 1 [file DataSheet_1.pdf]

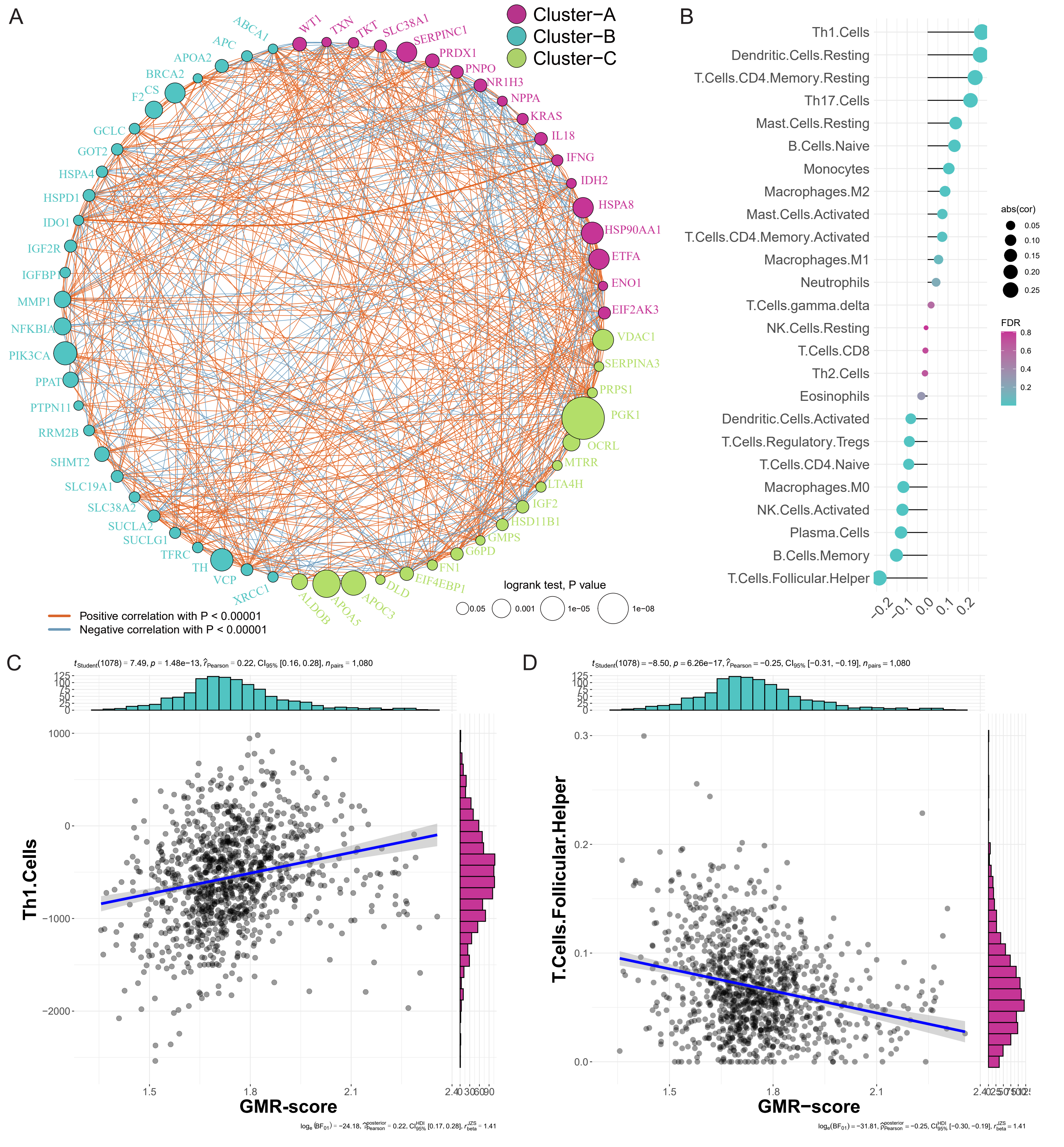

Figure S1. Relationship between GMR genes and TME.(A) Interactions among 63 prognostic GMR regulators. The circle size indicates the prognostic P value in BC patients. The interactions are represented by each line: blue denotes a negative correlation, whereas red denotes a positive connection. (B) The relationship between 25 immune cell and the GMR-score in combined dataset. (C) Correlation of GMR-score with Th1 cells. (D) Correlation of GMR-score with T Cells Follicular Helper.
