# Supplementary material for: Enhancing breast cancer outcomes with machine learning-driven glutamine metabolic reprogramming signature
Source: Front Immunol. 2024 May 1;15:1369289. doi: 10.3389/fimmu.2024.1369289 (PMC11097668; doi:10.3389/fimmu.2024.1369289)
Supplement: Supplementary file 2 [file DataSheet_2.pdf]

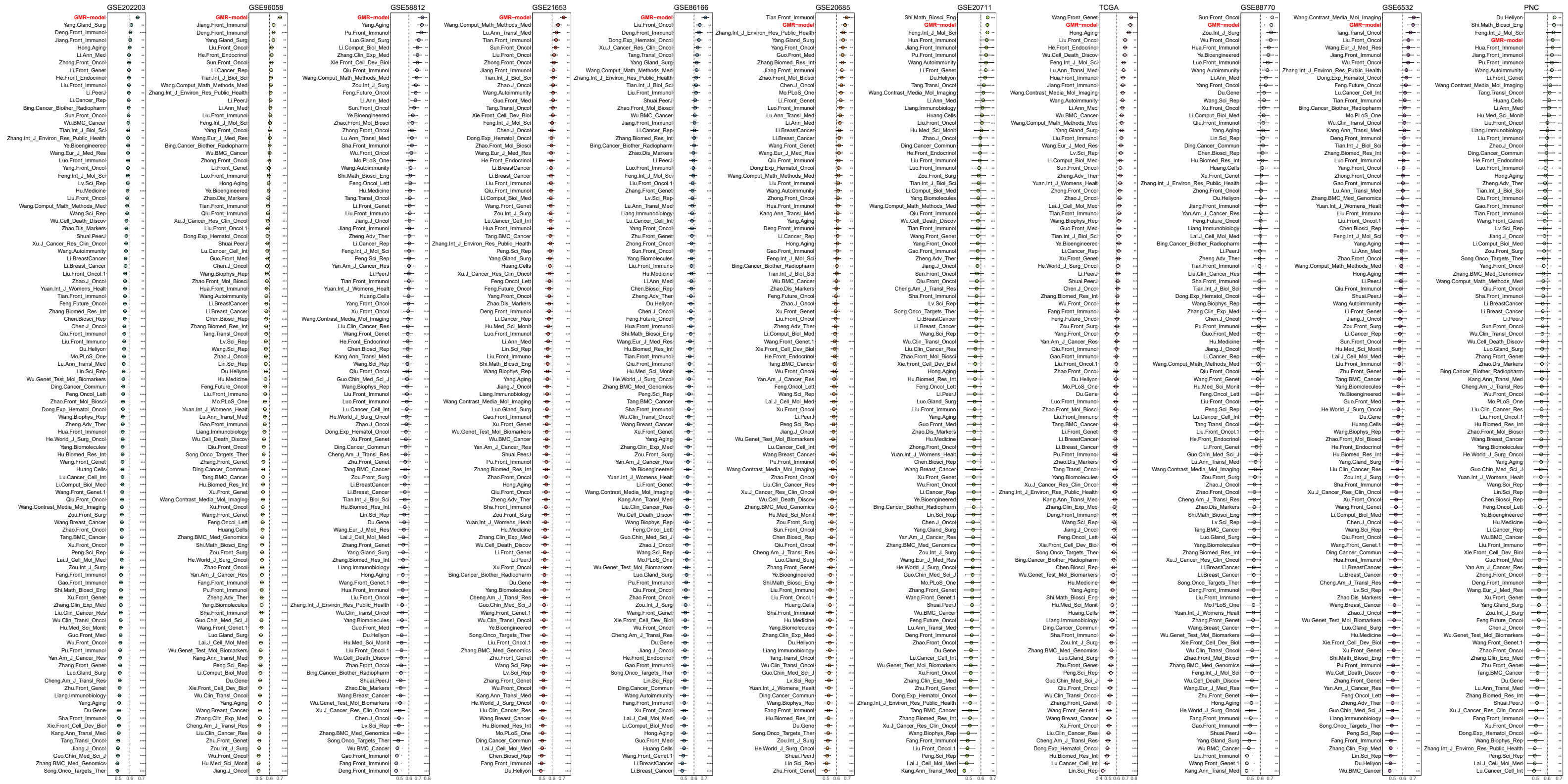

Figure S2. Comparing Cindex of GMR-model with 100 published signatures in the 11 BC cohorts.
